# The Design and Development of a Low-Cost and Environmentally Friendly Voltage Divider for On-Site High-Voltage Calibration up to 850 kV

**DOI:** 10.3390/s25133964

**Published:** 2025-06-26

**Authors:** Mohamed Agazar, Hanane Saadeddine, Kamel Dougdag, Mohamed Ouameur, Massinissa Azzoug

**Affiliations:** LNE (Laboratoire National de Métrologie et d’Essais), 1 Rue Gaston Boissier, 75015 Paris, France; hanane.saadeddine@lne.fr (H.S.); kamel.dougdag@lne.fr (K.D.); mohamed.ouameur@lne.fr (M.O.); massinissa.azzoug@lne.fr (M.A.)

**Keywords:** high-voltage calibration, voltage divider, modular divider, on-site measurements, impulses, direct current, alternating current

## Abstract

This paper presents the design, development, and characterization of a low-cost and environmentally friendly high-voltage divider optimized for on-site calibration up to 850 kV. Unlike traditional dividers that rely on oil or SF_6_ for insulation, both of which pose environmental risk and regulation issues, the proposed system uses modular construction with commercial off-the-shelf components and natural air insulation, minimizing environmental impact and facilitating transport, calibration, and maintenance. Despite using air insulation, the divider demonstrates excellent uncertainty performance. Characterization results show frequency linearity better than 0.2% up to 100 kHz and a bandwidth exceeding 10 MHz, making it suitable for the measurement of a wide range of voltage types. Static and dynamic performance evaluations confirm reliable scale factor stability and low measurement uncertainty: 0.01% for DC (550 kV), 0.3% for AC (405 kV), and 0.7% for impulses such as 1.2/50 µs (850 kV). The system offers a practical and sustainable solution for high-voltage measurements, meeting growing industrial and European environmental demands.

## 1. Introduction

Electric power transmission and distribution systems have evolved significantly since the late 19th century to meet growing industrial and societal demands. Over the past two decades, these systems have undergone further transformation driven by rapid advancements in quantum technologies, digitalization, and the integration of renewable energy sources. These developments have not only reshaped the structure of modern grids but have also underscored the critical importance of accurate, traceable, and robust measurement systems to ensure the reliability, efficiency, and safety [1,2]. Such measurement systems must deliver high precision under challenging conditions. For direct current (DC) measurements, uncertainties better than 0.02% are typically required to ensure that overall system accuracy remains within 0.1% [3]. For alternating current (AC) and impulse measurements, uncertainties below 1% are generally needed to guarantee accuracy levels within 3% [4].

To meet these accuracy requirements, regular calibration of high-voltage measuring equipment is essential. This calibration not only minimizes measurement errors but also ensures consistency with national and international references. It supports compliance with standards such as IEC 17025 [5], enhances confidence in measurement data, and reduces the risk of safety issues or product nonconformity. As the National Metrology Institute (NMI) in France, the LNE (Laboratoire National de Métrologie et d’Essais) is responsible for providing calibrations, tractable to international units (SI), of such quantities. Given that much of the high-voltage equipment is large, fixed, or integrated into the industrial infrastructure, on-site calibration is often the only viable option. A key component in these calibration systems is the high-voltage divider, which scales down voltages to levels compatible with standard measurement instruments such as voltmeters, digitizers, or oscilloscopes. The accuracy of the overall measuring system heavily depends on the performance of the divider, which must be specifically designed to handle the characteristics of voltage, such as DC, AC, or impulse.

However, conducting on-site calibrations using traditional voltage dividers has become increasingly difficult due to environmental regulations and concerns. The existing dividers [6] rely on insulating substances like sulfur hexafluoride (SF_6_) or oil, which, although effective electrically, pose serious environmental risks. SF_6_, for instance, is one of the most potent greenhouse gases with a global warming potential thousands of times higher than CO_2_. Similarly, oil-based systems involve risks of leakage, contamination, and complex disposal procedures. Regulatory frameworks, particularly in Europe [7], are moving toward the restriction and eventual elimination of these substances from high-voltage equipment. This shift is driven by a growing need to align industrial practices with global climate and sustainability goals. The regulatory pressure, combined with growing client reluctance to host gas or oil-insulated devices on their sites, underscores the urgent need for more sustainable measurement solutions at very high voltages.

In response to these new environmental needs, we have initiated the development of a new voltage divider, designed specifically to enable high precision on-site measurements while aligning with environmental standards. Similar work has been conducted in the past [8], but for voltages limited to only 200 kV. By eliminating the use of harmful insulating media, our new approach seeks to balance measurement performance at voltages much higher than 200 kV, operational practicality, and environmental responsibility. Nevertheless, this transition presents challenges as it can lead to a degradation in measurement accuracy compared to SF_6_ or oil-based dividers, especially those developed recently by NMIs [9,10]. Transitioning to air-insulated dividers is a necessary evolution to meet future emerging environmental standards and to enable calibration operations on-site, especially in sensitive industrial environments.

This paper presents the design methodology, characterization results, and uncertainty analysis of a new voltage divider. The approach developed by LNE aims to meet the growing demand for on-site high-voltage calibration systems that are both technically reliable and environmentally sustainable, particularly in challenging industrial applications [11,12,13], where voltages may reach up to 4 MV for impulse, 2.5 MV for DC, and 1.2 MV for AC. These levels require calibration capabilities, at lower levels, up to 800 kV, 500 kV, and 240 kV, respectively, based on the extrapolation factor of 5 specified in [2] and validated in the 19ENG02 project [14]. The developed divider exceeds these requirements, achieving voltage levels of 850 kV for impulse, 500 kV for DC, and 405 kV for AC.

Our prototype divider, built using natural insulation and with cost-effective components, achieves measurement uncertainties suitable for a wide range of calibration needs. We have shown that, with a careful component selection, mechanical design, and modular architectures, it is possible to achieve very good measurement performance.

## 2. Theoretical Framework and Background

**Figure 1:**
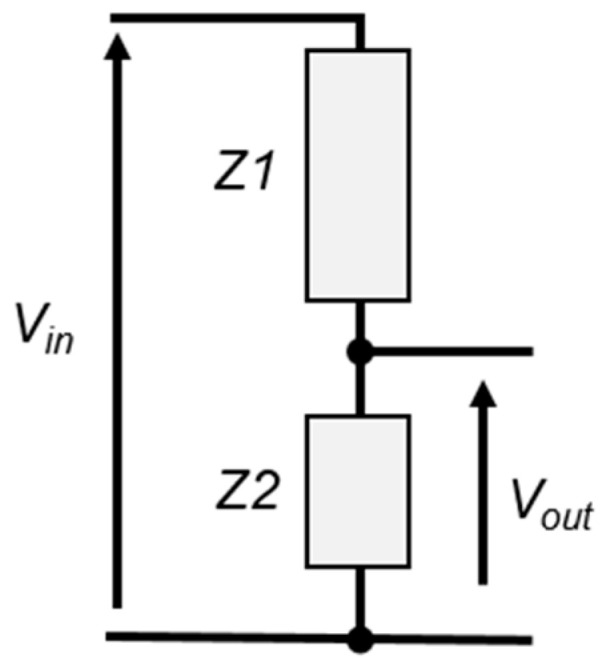
The principle of a high-voltage divider.

The most basic device in a high-voltage measurement chain is the voltage divider, Figure 1.

It consists of two impedances *Z*1 and *Z*2 connected in series. The input voltage *V_in_* is proportional to the output voltage *V_out_* based on the impedance ratio according to Equation (1).

(1) $$V_{in}=\frac{Z1+Z2}{Z2}\cdot V_{out}$$

This device acts as a voltage-to-voltage converter, reducing the voltage *V_in_* to a much lower voltage *V_out_*, typically below 1000 V, so that it can be safely measured using conventional instruments such as voltmeters, acquisition cards, oscilloscopes, or digitizers. Usually, a high-voltage divider is characterized by its scale factor (*SF*), or transfer function, which is the ratio between *V_in_* and *V_out_* according to Equation (2). The scale factor of a divider indicates how much the input voltage is scaled down.

(2) $$SF=\frac{V_{in}}{V_{out}}=\frac{Z1+Z2}{Z2}$$

Several types of voltage dividers are used in high-voltage measurements. The selection and configuration of their impedances (*Z*1 and *Z*2) must be matched to the type of voltage being measured. Voltage transformers are common in power systems and offer high accuracy at low frequencies (15–400 Hz), better than 0.01% at 50 Hz, and calibration methods down to 0.002% [15]. Resistive dividers are preferred for precise DC measurements, achieving better than 0.001% accuracy [16] and capable of capturing fast transients like 1.2/50 µs with 0.5% accuracy when optimized [17]. Capacitive dividers are mainly used for AC measurements up to several tens of kilohertz, with compressed gas types reaching 0.001% accuracy under strict temperature control [18], while other types struggle to achieve better than 0.01%. The damped capacitive divider improves impulse measurement performance by adding a resistor to suppress high-frequency oscillations while maintaining standard AC accuracy. Mixed RC dividers combine resistive and capacitive branches in parallel and, with proper compensation, can reach bandwidths of several tens of MHz, making them suitable for both DC and AC applications. Finally, the universal divider includes two branches: a high-ohmic resistive branch for DC in parallel with a damped capacitive branch. Its key strength is the ability to measure all voltage types with good accuracy, including DC, AC, harmonics, supraharmonics, transients, disturbances, and combined waveforms. However, thermal interactions between branches must be carefully managed to ensure high accuracy. This solution was selected for its versatility: with only a single divider, it becomes possible to measure all types of phenomena encountered in high-voltage applications. Very few universal dividers have been developed by NMIs. Table 1 presents two voltage dividers recently developed by National Metrology Institutes (NMIs). Divider 1 [9] uses SF_6_ insulation and is rated for impulse voltages up to 1400 kV. Divider 2 [8], by contrast, uses air insulation but is limited to voltages up to 200 kV. The measurement uncertainties reported for both systems are consistent with the levels targeted in our work.

**Figure 2:**
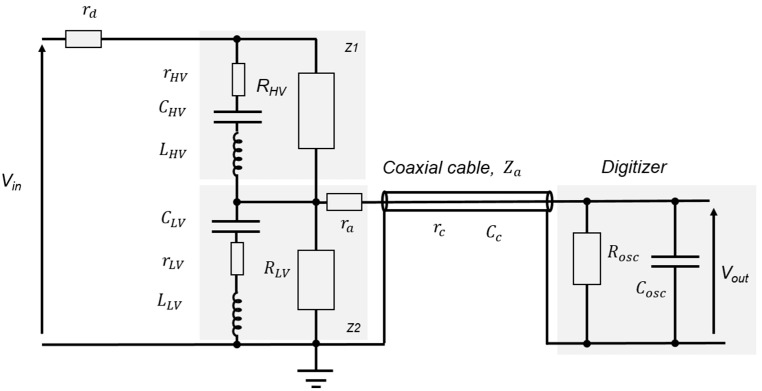
Design of the high-voltage divider based on the Zaengl divider principle.

The universal divider is based on the Zaengl principle [19], as described in Figure 2. *R_HV_*, *C_HV_*, and *r_HV_* constitute the impedance *Z*1, *r_d_* is an additional external damping resistor, and *L_HV_* is its total inductance (inductance of components and connections). *R_LV_*, *C_LV_*, and *r_LV_* compose the impedance *Z*2. *L_LV_* is its total inductance. The divider is connected to a digitizer, with its input impedance *R_osc_*//*C_osc_*, using a coaxial cable having a characteristic impedance *Z_a_* (*r_c_* is its total resistance, *C_c_* is its total capacitance with neglecting its total inductance). To avoid reflections on the coaxial cable, a damping resistor *r_a_* is added. Its value is approximately equal to the characteristic impedance of the cable.

For distortion-free measurements and to have optimum transfer in the divider, the time constants of the impedance *Z*1 and the time constants of the impedance *Z*2 must be matched. The value of components needs to be chosen to fulfill approximately the following conditions, Equations (3)–(5):

(3) $$R_{HV}\cdot C_{HV}\approx R_{LV}//(R_{osc}+r_{a}+r_{c})\cdot (C_{LV}+C_{c}+C_{osc})$$

(4) $$C_{HV}{\cdot (r}_{HV}+r_{d})\approx C_{LV}{\cdot r}_{LV}$$

(5) $$\frac{C_{HV}}{L_{HV}}\approx \frac{C_{LV}}{L_{LV}}$$

The non-inductive design prevents oscillations caused by the *RLC* resonant circuit. For effective damping, the damping resistor can be calculated [20] using Equation (6)

(6) $$r_{HV}+r_{d}=1\;to\;2\times \sqrt{\frac{L}{C}}$$

where *L* is the total inductance of the impedance *Z*1, and *C* is its total capacitance. In practice, due to the difficulty of determining the total inductance of *Z*1 with low uncertainty, the damping is implemented using a combination of internal and external resistors. The internal damping is achieved with the internal resistor *r_HV_*, while the external damping is achieved with an external resistor, which is determined with experimentation (step response technique).

The most critical parameters for achieving high accuracy with a voltage divider are the voltage coefficient (*VC*), temperature coefficient (*TC*), and the self-heating. The *VC* represents the relative deviation of the impedance when measured at 100% ($Z_{100\%})$ and 10% ($Z_{10\%})$ of the rated voltage using Equation (7).

(7) $$VC(\%)=\frac{{Z_{100\%}-Z}_{10\%}}{Z_{10\%}}\times 100$$

The *TC* represents the relative deviation of the impedance *Z_T_*_1_ measured from the reference temperature *T*1 and the measured resistance Z*_T_*_2_ at the maximum operating temperature *T*2, calculated according to Equation (8). It should be noted that this formula is only valid if the variation between the two temperatures is approximately linear, which is often the case over small temperature intervals.

(8) $$\mathrm{TC}(ppm/{}^{\circ}C)=\frac{\left(Z_{T2}-Z_{T1}\right)}{Z_{T1}\cdot (T2-T1)}\times 10^{6}$$

The self-heating can be characterized by the relative deviation of the impedances *Z_t_*_1_ and *Z_t_*_2_ between two time points, *t*1 and *t*2, respectively, as defined in Equation (9). Unless otherwise specified, time *t*1 is generally set to 1 min and *t*2 is set to 10 min.

(9) $$Self-heating(\%)=\frac{\left(Z_{t2}-Z_{t1}\right)}{Z_{t1}}\times 100$$

The most critical impedances for ensuring good accuracy are those subjected to high voltage. These include the high-voltage resistors (*R_HV_*), the high-voltage capacitance (*C_HV_*), and the damping resistor (*r_HV_*), which need to be selected with care.

## 3. Design Methodology

### 3.1. Selection of the DC Branch (R_HV_)

To minimize the influence of the leakage current of the resistor *R_HV_*, we decided to maintain the DC current at a high level of 0.5 mA. Under these conditions, the use of a shielding technique to eliminate the leakage current is unnecessary. However, the influence of self-heating and the influence of dissipated power must be carefully characterized.

**Figure 3:**
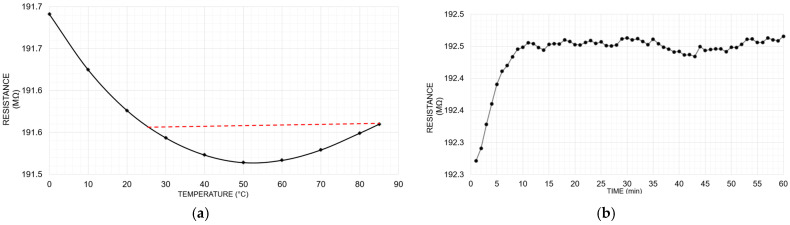
Characterization results of 50 kV tick film resistors; (**a**) temperature dependence from 0 °C to 85 °C, showing a nonlinear behavior compared to the declared *TC* (red line); (**b**) time dependence from 1 min to 60 min at 50 kV.

Thick film resistors are commonly used in the field of high-voltage applications as they can handle extreme voltages up to several tens of kilovolts in air insulation. They are also specified to operate over a wide temperature range (e.g., from −55 °C to +225 °C). We have selected and measured the *TC*, self-heating, and *VC* of several thick-film resistors rated at 50 kV/200 MΩ. Figure 3 presents the typical results obtained from these measurements. Selected with a *TC* of 10 ppm/°C, it appears very suitable, but measurements from 0 °C to 85 °C reveal that its variation is not linear. Most resistor manufacturers calculate the *TC* by measuring only the difference between two specific points (typically 25 °C and 85 °C) represented by the red line in Figure 3a, sometimes leaving out information about the resistor’s behavior between these two points. The red line clearly shows a *TC* of 10 ppm/°C between 25 °C and 85 °C, which is consistent with what is declared by the manufacturer, but in reality, the temperature dependence between 25 °C and 85 °C is outside this value.

The results also indicate that these resistors exhibit significant *VC* in air insulation, with a resistance variation of approximately 0.1% from 5 kV to 50 kV (Figure 3b), which is mainly due to the temperature rise within the resistive element of the resistors. The curve shows that their resistance increases steadily before stabilizing. This behavior is due to the internal temperature rise that only reaches a steady state after 10 min. The temperature increase caused by the applied voltage in air insulation makes it challenging to achieve high accuracies. This temperature rise directly affects both the VC and the self-heating behavior.

We decided then to use a set of a great number of low-voltage resistors (e.g., 500 V) assembled in series instead of a small number of high-voltage resistors (e.g., 50 kV). Low-voltage resistors typically exhibit a minimal *VC* and a linear *TC* over a wide temperature range. Such characteristics can be found in wire wound resistors, metal film resistors, or ultra-stable foil resistors. Our choice was on metal resistors as they are more readily available in high ohmic values (e.g., >1 MΩ) compared to wire wound resistors, which are only in low ohmic values (e.g., <100 kΩ). They are also relatively 20 to 30 times cheaper than ultra-stable foil resistors.

Resistors of 1 MΩ ± 1% from Vishay, type CMF60, 1 W, 500 V, were selected for their excellent characteristic in withstanding the stress generated by very fast impulses. The *VC* of these resistors have been measured to be 0.016%. At a rated voltage, the self-heating is only 0.0005%/min, which means that applying the rated voltage for up to 10 min results in a resistance variation of about 0.005%. The T.C is around 25 ppm/°C, which is sufficient to achieve a good accuracy.

Other resistors were also tested: metal electrode leadless face (MELF) resistors from Vishay, 1 MΩ, 300 V, 250 mW, 15 ppm/°C, exhibited better performance with a *VC* of 0.005% and self-heating of 0.001%. However, we decided not to use these resistors because we have discovered that they are sensitive to electric fields. Indeed, we have developed a 50 kV section by assembling 200 resistors in series on a printed circuit board (PCB). Despite the excellent characteristics of each individual component, it turned out that applying a high voltage generates an intense electric field around the resistors, which influences their *VC*. We have observed a *VC* of 0.1% of the 50 kV section instead of 0.005% when considering the *VC* of each resistor individually.

Resistors from Vishay, type FPT65, 1 MΩ, 1%, 10 ppm/°C, 250 mW have been also tested. They demonstrated a good performance, with a *VC* identical to the *VC* of the CMF60 type, but with a better self-heating (<0.0005%). Despite their excellent characteristics, these resistors are approximately five times more expensive than the CMF60 and are only available with very long lead times, which influenced our decision not to consider them.

### 3.2. Selection of AC and Impulse Branches (C_HV_ and r_HV_)

The total capacitance of *C_HV_* needs to be in the range of 200 pF to 1 nF to minimize the impact of parasitic capacitances to ground and to limit the power dissipation by the voltage source during AC on-site measurements. The capacitor and the damping resistor must be selected carefully. The capacitor must be non-inductive and capable of handling very high currents. Similarly, the damping resistor *r_HV_* must also be non-inductive and capable of withstanding very high voltages. Indeed, during very fast transient phenomena (e.g., 1.2/50 µs), the impedance of the capacitor becomes equivalent to a short circuit at high frequencies. In such cases, a very high peak current flows through the capacitor, and a significant portion of the voltage is absorbed by the damping resistor. The capacitor then needs to handle a very high peak current, and the damping resistor needs to handle a very high peak voltage and peak power.

**Figure 4:**
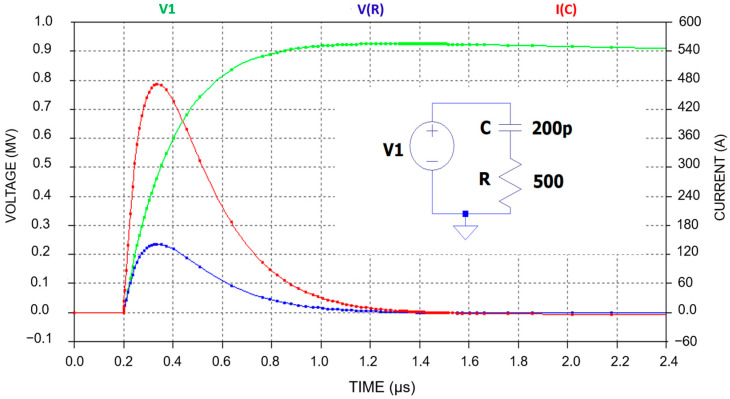
A simulation of the capacitive branch of the divider for impulse voltage up to 900 kV. The HV capacitor is simulated with a value of 200 pF, and the damping resistor is simulated with 500 Ω. The green curve represents the voltage to be measured, the red curve represents the current flowing through the divider, and the blue curve represents the voltage across the damping resistor.

The current level that the capacitor must withstand and the voltage across the damping resistor, during the application of a 1 µs rise time pulse with an amplitude of 900 kV, are simulated and shown in Figure 4. The pulse divider is modeled as an *RC* circuit (neglecting the inductance) with a total capacitance of 200 pF and a total damping resistance of 500 ohms. The current through the capacitor spikes sharply to around 500 A at the initial application of voltage. The current then decreases exponentially as the capacitor charges, approaching zero as the voltage across the capacitor stabilizes. The voltage across the resistor follows the same behavior as the current. It peaks early in the process and then decreases exponentially as the capacitor charging completes. The simulation shows that the capacitor must handle a peak current of approximately 480 A (red curve), and the voltage across the resistor reaches approximately 250 kV (blue curve).

For impulse measurements, the capacitor needs to have a very good frequency and voltage linearity to avoid distorting the impulse wave shape to be measured. A good *TC* is also needed in order to handle its temperature rise during impulse measurements. Indeed, the application of the impulse will produce a localized temperature rise that can reach several tens of degrees Celsius due to the high current flowing through the capacitor and the damping resistor. For AC measurements, a good *VC* and a good self-heating effect are needed to achieve high accuracy when measuring the AC voltage.

The number of capacitors that meet these requirements is very limited. Capacitors with excellent performance for AC measurement have very low capabilities for handling high currents, while capacitors capable of absorbing high currents have poor AC characteristics. There are two types of capacitors that can meet our needs: negative/positive-zero (NP0) ceramic capacitors, known for their very low *TC* (<30 ppm/°C) but also known for being highly fragile to vibrations and transport. Polypropylene capacitors have a higher *TC* of around (e.g., <300 ppm/°C) but are reputed for their long-term stability, better durability, and their ability to withstand vibrations during transport. Since the divider used in this study will often be transported, we have chosen the second solution.

Polypropylene film capacitors type FKP4 from Wima (Folien Kondensator Polypropylen) have been selected for their small size compared to the FKP1 type. They have been chosen for their high dV/dt rating (high current peak withstanding). Capacitors of 0.1 µF/1000V have been selected; their dV/dt is equal to 9 kV/µs, and the maximum admissible current is then equal to C.dV/dt = 900 A. These capacitors have the ability to withstand 1.5 times of nominal voltage over short durations (e.g., 1.2/50 µs).

Temperature, voltage, and frequency dependencies of the capacitors have been checked to verify the values declared by the manufacturer. The frequency dependence of the capacitors was measured using a calibrated HP 4284A RLC meter. These measurements were performed at a low voltage (a few volts), allowing precise evaluation of the capacitance over a range of frequencies up to 100 kHz. To assess the temperature dependence, the capacitors were placed in a high-precision, temperature-controlled chamber, and measurements were carried out across a temperature range from 10 °C to 30 °C. This ensured a reliable characterization of thermal stability. The voltage dependence was investigated at 50 Hz by applying increasing AC voltages up to 600 V RMS. The applied voltage was measured using a calibrated voltmeter, and the current through the capacitor was recorded to determine the apparent capacitance at each voltage level. These methods allowed for a thorough verification of the values declared by the manufacturer under conditions representative of practical use.

**Figure 5:**
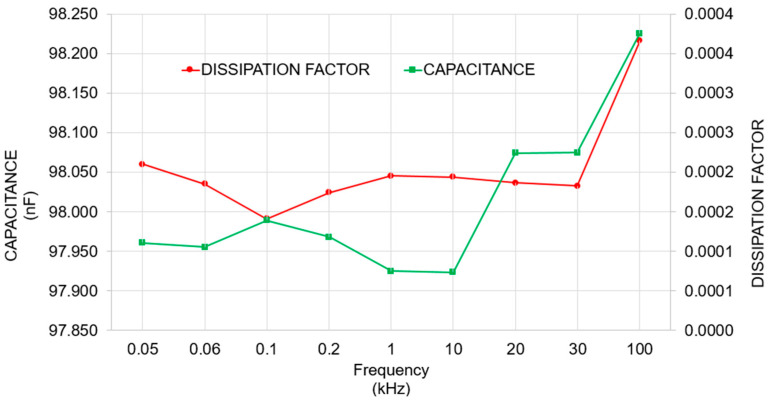
Frequency dependence (capacitance value and dissipation factor) of the selected capacitor from 50 Hz to 100 kHz.

The capacitors demonstrate good capacitance stability and a linear *TC* around −200 ppm/°C, measured from 10 °C to 40 °C. The typical influence of frequency is shown in Figure 5, conducted over frequencies ranging from 50 Hz to 100 kHz. The capacitance remains relatively stable for most of the lower frequency range up to 10 kHz, with only minor fluctuations. Beyond 10 kHz, there is a noticeable increase in capacitance of about 0.3%, with the values rising sharply, potentially caused by changes in dielectric properties. The dissipation factor starts near zero and remains minimal. Beyond 30 kHz, it rises sharply, indicating increased energy dissipation or losses, but still lower than 0.0004. The *VC* of some capacitors has been checked up to 600 V rms; typically measured to be in the range of 0.3%, which appears to be a good compromise. These capacitors are capable of withstanding their rated voltage up to at least 2 kHz, then decrease significantly, taking into account the admissible rms current that they can handle. By using these capacitors, the final divider will be capable of measuring relatively high frequencies up to 2 kHz at a maximum voltage, even though calibration requests are absent at such high frequencies.

For the damping resistor *r_HV_*, we have chosen to connect a resistor in series with each capacitor. Assuming that each capacitor has an inductance of 100 nH, including the connections, Equation (6) gives a required resistance value of 1 Ω. Resistors of type WNB1R0FET from OMITHE have been selected for their high pulse capability. They are rated at 1 W and can withstand five times the rated power (i.e., 5 W) for 5 s. This means that the maximum energy these resistors can absorb is 5 W × 5 s = 25 J. Since the resistors are subjected to a maximum voltage of 500 V (when the series capacitor-resistor pair is exposed to a 1500 V pulse), the resulting absorbed energy is approximately 0.25 J during approximately 1 µs. For other longer impulses, the voltage at the terminals of the damping resistors decreases.

### 3.3. Electrical and Mechanical Design

**Figure 6:**
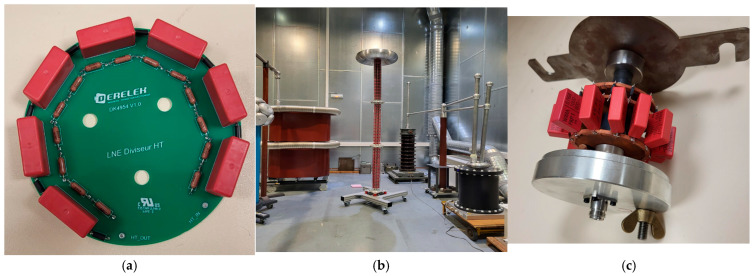
The mechanical development of the divider: (**a**) the 7 kV section designed using FR4 PCB boards. Each section contains 7 capacitors, 7 damping resistors, and 14 DC resistors. (**b**) The final divider assembly consists of 3 modules, each module containing 27 sections arranged in series inside a Plexiglas tube. (**c**) The impedance *Z*2 is designed in a coaxial structure and is fixed at the bottom, beneath the base of the divider.

Sections of 7 kV each have been designed in PCB FR4 boards (Figure 6a). Each section contains 7 capacitors, 7 damping resistors, and 14 DC resistors. The precision components are arranged in a circular pattern, ensuring uniform voltage distribution. The symmetrical arrangement of the components helps maintain electrical stability and thermal balance. The layout follows best practices for high-voltage insulation, with clearance distances to prevent arcing. A 300 V/mm dielectric strength, proprietary to the PCB, taking into account its aging, has been used to fix the minimum distances between components to avoid possible flashover. The distances were doubled to allow each section to operate at 1.5 times its nominal voltage, i.e., 10.5 kV for impulse measurements. The HT_IN and HT_OUT terminals indicate designated input and output points. Three holes are used to insert threaded rods to fix the sections.

To reduce the weight of the divider and for environmental reasons explained in the introduction, we have chosen not to use oil or gas for electrical insulation. Instead, we decided to implement a divider with natural insulation (air). This choice comes with consequences as the use of air introduces an additional level of complexity in managing heat dissipation. Oil, with its thermal inertia and the same equivalent volume, has the advantage of maintaining a certain temperature homogeneity inside the divider. In contrast, the temperature of the air-insulated divider will be influenced by the heat generated by the components during the application of voltage and by the thermal exchange between the components and the external environment.

Three modules have been developed. Each one consists of 27 sections in a vertical configuration. The design has been chosen to prioritize compactness and coaxial alignment to reduce inductance. The sections are mounted in series inside a Plexiglas tube. The distance between the sections is fixed at 35 mm using epoxy spacers. The three modules have been cascaded in series to construct the final divider (Figure 6b). We used three corona rings to reduce corona effects. For the first and second modules, we used two corona rings with a diameter of 500 mm and a tube size of 125 mm. For the last module, we used one corona ring with a diameter of 740 mm and a tube size of 180 mm. These corona rings, in addition to the coaxial geometric arrangement of the sections, significantly reduce corona effects.

To satisfy Equations (2) and (3), we developed the impedance *Z*2 using the same components as the impedance *Z*1 (Figure 6c). This approach helps compensate for the effects of frequency, voltage, and temperature. Each individual element of the impedance *Z*2 could be subjected, for better matching, to the same voltage and current constraints as each element of the impedance *Z*1. We have then selected the components with the same technologies used in the sections. DC resistance type CMF60, capacitors type FKP4 from Wima, and damping resistors type WNB from Ohmite have been used. Of course, the values will differ from those of the sections, which will result in the compensation system being moderately effective. To satisfy Equation (5), achieving correct compensation for the inductance of the *Z*1 is very challenging as it is difficult to estimate accurately. It is not necessary to minimize the inductance entirely, but rather to ensure that the equation is satisfied. For example, if the inductance of *Z*1 is estimated at 10 µH, and the divider has a ratio of 1000, the inductance of *Z*2 must be equal to 10 nH. The lower the voltage scale factor, the more the condition can be satisfied. This is also favorable because for smaller scale factors, the signal-to-noise ratio is better. Indeed, during a transient phenomenon, transmitted electromagnetic disturbances can be superimposed on the signal transmitted through the coaxial cable, which can influence the accuracy of measurement. The final divider has been adjusted to a scale factor of 1000 by several iterations to satisfy Equations (3)–(6). An external damped resistor of 200 Ω has been used.

A chassis has been developed with appropriate elements to fix the tubes. The impedance *Z*2 is fixed on the lower side of the chassis, and the modules are fixed on the upper side. Indeed, each module has been equipped with a flange on both sides, and the modules and corona rings could be fixed using these flanges. The flange features multiple holes arranged in a concentric pattern. The outer circle has evenly spaced holes for bolting the modules together. The inner holes are used to fix a threaded rod supporting the sections.

At the output of *Z*2, a shielded triaxial cable with a 50 Ω impedance, and a capacitance of 2 nF is used to connect the divider to a digitizer with an input impedance of 30 pF//1 MΩ. This digitizer is exclusively used for impulse measurements. For AC measurements, it is replaced by a voltmeter with an input impedance of approximately 40 pF//1 MΩ. For DC measurements, a precision voltmeter with input impedance of 10 MΩ in parallel with an additional external resistor of 1.111 MΩ is used. In this case, the equivalent resistance in the output of the divider at DC is still 1 MΩ. Table 2 summarizes the rated voltage that can handle the voltage divider.

The nominal impulse voltage of 850 kV has been derived from the accumulated nominal values of each section of the divider, with each section rated for 10.5 kV under 1.2/50 µs impulse conditions. The final configuration includes 81 sections (3 modules × 27 sections), resulting in a total rated voltage of 81 × 10.5 kV = 850.5 kV. Direct testing at 850 kV was not feasible in our current facility. Therefore, the performance of the divider was first verified at lower voltages (e.g., up to 350 kV) by comparison with our reference system [16]. No significant deviation in the scale factor was observed within this voltage range. To support the extrapolation to 850 kV, we relied on the characterization of a single 10.5 kV section, whose linearity was measured from 1 kV to 10.5 kV. This extrapolation technique [1,2] is commonly used in the high-voltage field when no standard is available at such voltage levels. An extrapolation factor of 5 is allowed.

### 3.4. Environmental Considerations

The developed voltage divider does not utilize a hermetically sealed enclosure, but instead, it relies on natural air insulation within an open Plexiglas (PMMA) tube structure. It is specifically designed for operation in controlled environments, either on-site or at calibration facilities, where ambient temperatures are maintained between 10 °C and 30 °C and relative humidity remains below 80%. Under these conditions, the influence of environmental factors on electrical performance is minimal.

Several measures were implemented to ensure stability and robustness under these operational conditions. The resistors and capacitors used are industrial-grade, encapsulated components selected for their low sensitivity to environmental factors such as humidity and temperature. These choices contribute to the system reliability in metrology laboratories and semi-industrial settings. To ensure scale factor stability during use, particularly in on-site applications, the calibration procedure includes a verification step before and after each high-voltage measurement, using a reference system at a low voltage (<100 kV), as described in Appendix A. This approach allows for early detection of any short-term drift caused by environmental or operational effects.

The mechanical structure employs FR4 PCB boards, epoxy spacers, and commercial polypropylene capacitors, materials selected for their proven electrical and mechanical performance in high-voltage systems. Although these materials are not specifically designed for harsh field conditions, they have shown good resilience under repeated high-voltage stress in controlled environments. For example, the polypropylene capacitors used in the high-voltage arm were subjected to over 1000 impulses (1.2/50 µs, 10 kV) during a component-level testing without measurable degradation. Similarly, the FR4-based sections and epoxy spacers showed no visible mechanical or dielectric deterioration.

That said, long-term environmental exposure remains a relevant consideration. FR4 may absorb moisture in high-humidity environments, potentially altering dielectric behavior, while epoxy spacers can degrade under thermal cycling. To mitigate these risks, the divider is operated strictly within its recommended environmental range and is stored and transported using UV-resistant and humidity barrier enclosures.

Our previous experience with smaller air-insulated dividers using similar Plexiglas constructions demonstrated long-term mechanical and thermal stability over several years of use. No significant performance deviations were observed under typical laboratory conditions. These findings support the suitability of this construction approach for higher voltage levels, provided standard environmental controls and handling precautions are observed. Although formal accelerated aging and endurance tests were not conducted as part of this study, the performance of the divider under these conditions could be planned for future work to further assess the durability of the materials and verify long-term reliability.

## 4. Experimental Characterization

### 4.1. Frequency and Impulse Response

According to our procedure, the scale factor of the voltage divider is typically determined up to 100 kHz at voltages above 100 V to ensure sufficient resolution while keeping the voltage below 1000 V. This limitation is mainly due to the constraints of the available equipment, such as the Fluke 5720 calibrator (Fluke Corporation, Everett, WA, USA), which cannot generate high frequencies at elevated voltages, and the physical height of the divider, which prevents the use of other instruments such as frequency analyzers. These analyzers are limited both by their maximum input voltage (typically around 10 V) and by environmental influences such as cable length and proximity effects (e.g., stray capacitances). For frequencies above 100 kHz, alternative techniques are more appropriate as explained below. The scale factor is determined by simultaneously measuring the input and output voltages: the input is applied using the calibrated Fluke 5720 (uncertainty <0.01%), and the output is measured using a calibrated voltmeter of a similar accuracy. This scale factor, measured up to 100 kHz, serves two purposes: it verifies the frequency response with respect to harmonics and supraharmonics, and it enables long-term monitoring of stability through annual calibration and drift checks performed before or after each measurement. The detailed on-site calibration procedure is provided in Appendix A.

The frequency linearity of the scale factor has been measured at 200 V from DC to 100 kHz. The results are presented in Table 3. A very good frequency linearity has been obtained with a flat frequency response within 0.2% up to 100 kHz. This is mainly due to the use of the same type of components in both impedances *Z*1 and *Z*2. This excellent linearity provides a reliable estimation of the scale factor if the divider is used to measure high-frequency phenomena (harmonics and supraharmonics up to at least 100 kHz).

For fast impulse measurements, performing a frequency sweep higher than 100 kHz is usually impossible due to the instrument’s limitation. However, we assess this using several methods:

- Time and frequency domains characterization;
- Analysis of the step response;
- Convolution technique.

**Figure 7:**
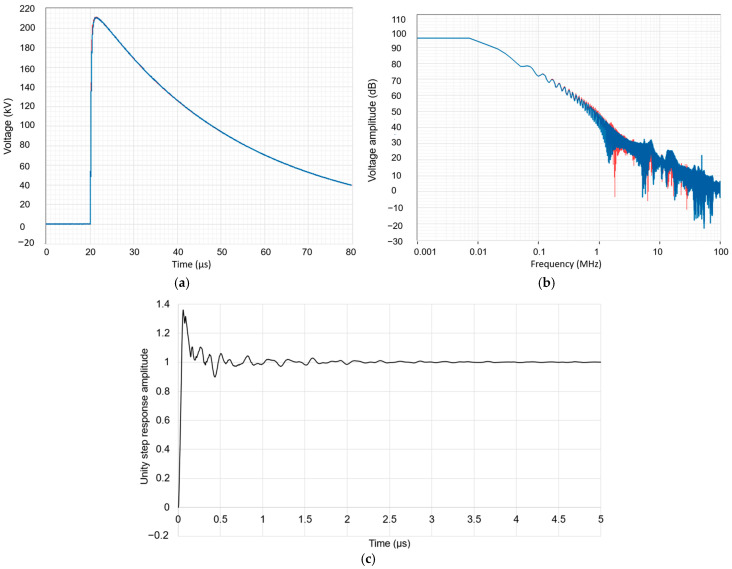
The time and frequency behavior of the voltage divider is evaluated through (**a**) the time-domain response to a 1.2/50 µs impulse of 210 kV, (**b**) the frequency-domain response to a 1.2/50 µs impulse of 210 kV, and (**c**) the unit step response, confirming a rise time of 35 ns and a 10 MHz bandwidth with stabilization observed from approximately 0.5 µs.

Figure 7a,b illustrate the performance of the high-voltage divider evaluated both in the time and frequency domains. In Figure 7a, the divider is subjected to a standard 1.2/50 µs lightning impulse with a peak amplitude of 210 kV. The voltage waveform is acquired at the output of the divider (red color), while the input reference signal (blue color) is measured using a standard high-voltage divider [16] with a known bandwidth of 35 MHz. The response is clean, with no significant overshoot or oscillation, indicating that the divider reproduces the overall time domain shape of the impulse with good fidelity. To investigate the frequency-dependent behavior, the fast Fourier transform (FFT) of both the input and output waveforms was computed and is shown in Figure 7b. Up to about 1 MHz, both spectra are nearly identical, indicating that the divider has a flat response and negligible amplitude distortion in this range. Beyond this frequency, the FFT magnitude of the divider begins to deviate from that of the reference. The difference becomes significant in the 1–20 MHz range, where the output signal of the divider attenuates more rapidly. The deviation becomes especially pronounced beyond ~10 MHz. It is important to note that this FFT comparison reflects the relative frequency response between the two dividers. Since the reference divider itself has a finite bandwidth (35 MHz), the measured frequency response of the divider may appear artificially better than it is beyond this limit. In other words, the FFT shows not the absolute frequency response of the divider but the difference between its response and that of the standard. Thus, the effective bandwidth of the divider can be estimated to be somewhat lower than 35 MHz, likely in the range of 10–15 MHz, although a more precise characterization would require step response analysis.

We have evaluated the dynamic performance of the divider, and its step response has been measured according to Annex C of IEC 60060-2 [2]. An external damping resistor of 200 Ω have been used during these tests to suppress oscillations on the front and to better satisfy Equation (5). Figure 7c shows the unit step response illustrating how the normalized amplitude evolves over time. A small high-frequency oscillatory behavior is observed, indicating minor overshoot and ringing, which suggests that the energy meter is adequately damped. The response stabilizes after approximately 0.5 µs, indicating that the system is capable of accurately measuring impulses with very fast rise times, such as 1.2/50 µs lightning impulses.

To validate the capability, especially for very fast impulses, we have used the convolution technique described in Annex D of IEC 60060-2 [2] to determine the errors of the divider for fast impulses with front times of 0.84 µs, 1.2 µs, and 1.56 µs, each with a total duration of 50 µs. The results are presented in Table 4. The peak voltage error is relatively small across all waveforms, ranging from 0.3% to 0.5%. Some variation is observed in the front time error, with the fastest waveform exhibiting the highest error at 2.4%, though this remains within acceptable limits.

### 4.2. DC and AC Performance

The characterizations at DC and AC/50–60 Hz have been performed for each phase before setting up the final divider. The characterization for each phase is important to ensure that the divider meets high accuracy. The most important parameters are the *VC* and self-heating. The *VC* measurements are presented as the relative deviation across voltage levels range from 10% to 100% of the rated voltage. The time behavior was assessed by applying the rated voltage for 10 min.

First, we have evaluated the voltage and time behavior of the individual sections. At DC, the sections exhibited very good voltage linearity with a maximum deviation of 0.006%. The time behavior was even better with a maximum deviation of 0.0005% over 10 min confirming its robustness for long-term voltage applications. For AC, as expected, the *VC* and self-heating effects were significantly higher, sometimes resulting in voltage deviations exceeding 0.6%, indicating a slightly higher sensitivity to voltage changes compared to DC. The time behavior showed a maximum deviation of 0.08% over 10 min, which is moderate compared with the *VC*.

The sections have also been tested by applying 1000 impulses 1.2/50 µs of 10 kV, with each impulse spaced 10 s apart. The objective was to test the insulation of the section and to verify the dielectric strength of all components (resistors and capacitors). After this, the DC and AC tests were remeasured once the components had returned to ambient temperature. The DC variations of sections before and after the application of 1000 impulses are extremely small, with a maximum deviation of 0.0002%, indicating excellent stability and excellent pulse capability of the selected resistors. The AC variations are more noticeable, with a variation of 0.07%, indicating slightly higher sensitivity of the sections compared with DC; however, it can be considered negligible, taking into account the large number of applied impulses spaced by only 10 s. Indeed, in routine calibration, the number of impulses is very limited, and the time spacing of the impulses is usually counted in minutes.

The sections have been validated, and their performance is in the same range as the results obtained with the individual elements, which confirms the predictability and calculability of our design. Indeed, by studying the behavior of individual elements, we can predict how the divider will behave. It appears that the influence of the electric field does not affect the performance of the individual elements, which validates our geometry.

Once the sections have been validated, we have then built three modules, each one containing 27 sections. We have evaluated the voltage and time behavior of the three developed modules. For DC, each module has been measured up to 185 kV by comparison with the LNE reference divider, which is capable of calibrating HVDC dividers with an uncertainty of 20 ppm. The modules exhibited very good voltage linearity, with a maximum deviation of 0.010%, and the time behavior showed a deviation of a maximum of 0.015% over 10 min. For AC, each module was measured by comparison with the LNE reference divider, which is capable of calibrating HVAC dividers with an uncertainty of 200 ppm. The *VC* was approximately 0.4% over the voltage range, and the time dependency was around 0.10% over ten minutes at the rated voltage. These results are consistent with those obtained with the section, which validates that our geometry is moderately sensitive to the electrical field.

**Figure 8:**
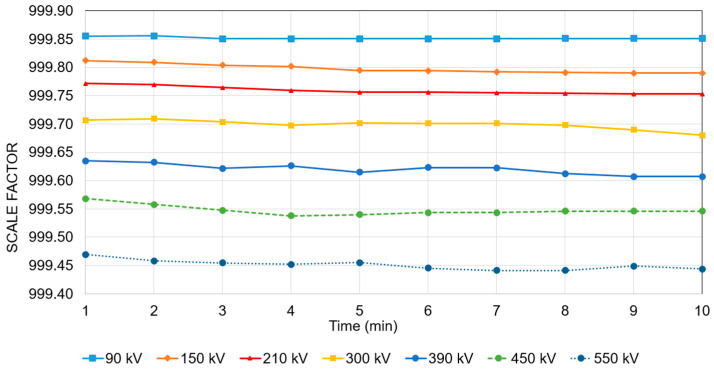
Variations of the scale factor of the divider against voltage up to 550 kV and time for DC.

Finally, the entire divider was tested at DC, and Figure 8 represents the *VC* of the scale factor of the divider and its variation over time. The parallel calibration method of the three modules was used to highlight the behaviour of the divider when the three sections are connected in series. The principle is that the final divider, at a maximum voltage, will be subjected to the same stresses as the three modules in parallel, but with one-third of the voltage. This method is used when a voltage reference is not available at the nominal voltage. The impedance Z2 was adjusted to achieve a ratio of 1000 with this parallel configuration.

The scale factor is plotted for different voltage levels (90 kV, 150 kV, 210 kV, 300 kV, 390 kV, 450 kV, and 550 kV) over 10 min. The curves show that the scale factor remains relatively stable, with only minor fluctuations over time for each voltage level. The stability of the scale factor over time is crucial for accurate high-voltage measurements, and the observed variations remain within a tight range (0.005%), indicating a well-performing voltage divider with a minimal time-dependent drift. The *VC* remains smooth, confirming a predictable change with voltage, which allows application of systematic corrections to reach an accuracy of at least 0.01%.

## 5. Uncertainty Analysis

The uncertainties are calculated in accordance with the Guide to the Expression of Uncertainty in Measurement (GUM) [21]. The methodology of the uncertainty calculation is described in IEC 60060-2 [2]. Table 5 summarizes an example of the obtained uncertainties for AC, DC, and impulses (e.g., 1.2/50 µs).

The voltage divider is intended to be used exclusively on-site. Once installed and assembled, the first step is to verify its voltage scale factor under calibration conditions to take into account errors such as proximity effects, drift, and temperature. For this purpose, a second small reference divider serves as the primary standard to calibrate the voltage divider at relatively low voltages (e.g., <100 kV). The obtained scale factor is extrapolated using the curves of the characterization results. For each measurement, AC, DC, or impulse measurements such as 1.2/50 µs, the main uncertainty contributions are listed below:

- Calibration uncertainty of the divider under real conditions using a second reference divider. Its standard uncertainty is estimated to be better than 0.0025% for DC, 0.025% for AC, and 0.25% for transients. The tractability of the measurement to international units is validated through international comparisons [22,23]. The calibration measurements and capabilities of LNE are described in [24].
- The influence of the *VC* has been assessed based on the results of characterization, using linear models. Its standard uncertainty is estimated to be better than 0.003% for DC up to 550 kV, 0.14% for AC up to 405 kV, and 0.12% for impulses up to 850 kV for 1.2/50 µs.
- The influence of the self-heating has also been assessed based on the results of the characterization using linear corrections. Its standard uncertainty is estimated to be better than 0.0014% for DC at 550 kV, 0.013% for AC at 405 kV, and negligible for transients.
- The influence of temperature can potentially be minimized by measuring the calibration and operating temperatures. This effect may be correlated with the two previously mentioned contributions. Considering a maximum temperature variation of 4 °C, we assume a *TC* around 25 ppm/°C for DC and 200 ppm/°C for AC and transients. The standard uncertainty is estimated to be better than 0.0029% for DC and 0.023% for AC and impulses.
- Other sources of uncertainty may arise, particularly those related to the digitizer, the instabilities of the sources, and the software used for processing the voltages. The standard uncertainty can be considered better than 0.001% for DC, 0.01% for AC, and 0.2% for impulses. For impulses, these sources of uncertainty have been determined using the procedures described in [25,26].

## 6. Discussion

The development of a high-voltage divider with air insulation instead of environmentally harmful substances like gas or oil is the best alternative to meet the regulatory requirements during transport or during the calibration itself at sensitive sites. Compared to traditional voltage dividers, the developed divider stands out from other precision or commercial dividers due to its simple and practical design; it uses readily available components, ensuring ease of sourcing worldwide with very reduced costs. Most of precision dividers that need high accuracy rely on expensive high-precision components and require long procurement times; our solution remains accessible to a much broader audience. In addition, its compact and lightweight structure allows for quick and easy on-site installation, without requiring heavy equipment like cranes or aerial platforms, thereby reducing the duration of the calibration and avoiding logistical constraints. This makes the divider highly portable without the need for special handling procedures.

A comprehensive characterization was performed at multiple levels: individual components, subsections, modules, and the complete system. The resulting measurement uncertainties are consistent with the precision expected in high-voltage applications. The divider satisfies the requirements for accurate measurement of DC, AC, and impulse waveforms. Due to its large bandwidth, it can also be used to accurately measure harmonics, supraharmonics (up to at least 100 kHz), and complex waveforms combining impulses and steady-state voltages.

The capacitors selected for the high-voltage arm are optimized for handling high peak currents and fast impulses. However, they are not ideal for AC voltage measurements when aiming for uncertainties comparable to those of a pure capacitive divider (e.g., <0.1%). Achieving such performance requires additional compensation for their voltage-dependent behavior on the low-voltage side. The solution to address this is to develop a dedicated low-voltage arm only for AC measurements, using capacitors that either compensate for the undesired voltage dependence or closely match the behavior of the high-voltage arm. This technique can be implemented experimentally by selecting and assembling low-voltage capacitors in a way that allows the low-voltage arm to emulate the characteristics of the high-voltage arm. This symmetry is expected to reduce the overall uncertainty by a factor of two or even three.

Our next objective is to increase the voltage capability of the divider up to 1.5 MV. This could be achieved by stacking three additional modules to reach the target voltage. However, this configuration would reduce the total capacitance to around 60 pF, which is insufficient to maintain high measurement accuracy due to the influence of parasitic capacitances to ground. To overcome this limitation, new developments have been initiated focusing on alternative structures, still using air insulation but increasing the capacitance of each section by a factor of 3 or 4. The aim is for the future 1.5 MV divider to have a total capacitance between 200 pF and 300 pF, ensuring accurate and stable performance for AC and impulses. The findings from this work will be presented in a forthcoming publication.

## Figures and Tables

**Table 1:** Universal reference dividers developed at the NMI level.

System	Insulation Type	Max Voltage (Impulse)	Uncertainty (DC)	Uncertainty (AC)	Uncertainty (Impulse)	Bandwidth
Divider 1	SF6	1000 kV	<0.01%	0.1%	0.5%	5 MHz
Divider 2	Air	200 kV	<0.1%	0.1%	0.5%	10 MHz

**Table 2:** Rated voltages that can handle the voltage divider.

	*R_HV_*	*C_HV_*	*r_HV_*	Maximum Voltage For:			
				DC	AC	1.2/50 µs	250/2500 µs
Section	14 MΩ	14.3 nF	7 Ω	7 kV	5 kV	10.5 kV	7 kV
Module	378 MΩ	529 pF	189 Ω	189 kV	135 kV	273 kV	189 kV
Energy meter	1.13 GΩ	176 pF	767 Ω	567 kV	405 kV	850 kV	567 kV

**Table 3:** Frequency response of the divider up to 100 kHz.

Frequency (kHz)	Scale Factor	Relative Uncertainty in % (k = 2)
DC	1003.5	0.01
0.06	1003.2	0.05
1	1003.2	0.05
10	1003.2	0.05
50	1003.6	0.05
100	1005.3	0.10

**Table 4:** Erros for the measurements of fast impulses.

Wave Shape	Voltage Peak	Front Time	Duration
0.84/50 µs	0.5%	2.4%	−0.5%
1.2/50 µs	0.5%	0.6%	−0.3%
1.56/50 µs	0.3%	1.3%	−0.2%

**Table 5:** Examples of calibration uncertainty for DC, AC, and fast impulses (1.2/50 µs).

	Uncertainty Sources	Uncertainty (%)	Coverage Factor	Standard Uncertainty (%)	Expanded Uncertainty (%) (k = 2)
DC 550 kV	Calibration	0.0050	2	0.0025	0.010
	Influence of voltage	0.0050	√3	0.0029	
	Self-heating	0.0050	√3	0.0014	
	Influence of temperature	0.010	2√3	0.0029	
	Others	0.0010	1	0.0010	
AC 405 kV	Calibration	0.05	2	0.025	0.30
	Influence of voltage	0.25	√3	0.14	
	Self-heating	0.02	√3	0.013	
	Influence of temperature	0.08	2√3	0.023	
	Others	0.010	1	0.010	
1.2/50 µs 850 kV	Calibration	0.50	2	0.25	0.68
	Influence of voltage	0.20	√3	0.12	
	Self-heating	0.00	√3	0.00	
	Influence of temperature	0.08	2√3	0.023	
	Others	0.20	1	0.20	

**Figure A1:**
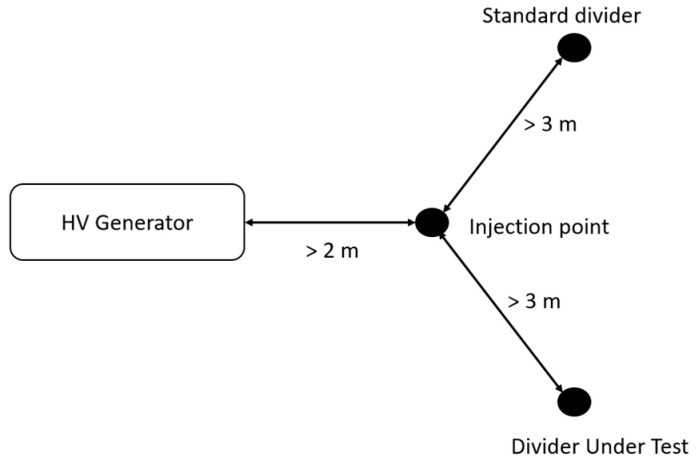
Reference configuration and minimum reference distances for impulse measurement.

**Figure A2:**
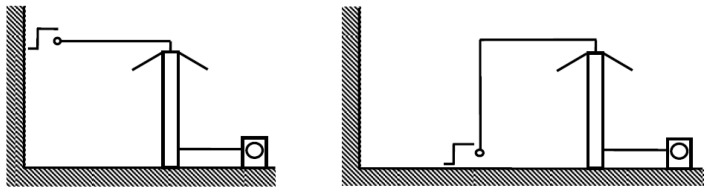
Performed setup for the step response measurement.

## Data Availability

The data presented in this study are available upon request from the corresponding author. The data are not publicly available due to ethical restrictions.

## Appendix A

### Procedure for Impulse Measurements

The voltage divider is the main component of the measurement system under calibration, but it cannot be calibrated independently. Given its usual use, it is necessary to define a consistent reference configuration (Figure A1) for its calibration, including high-voltage connection length, presence or absence of damping in the high-voltage loop, matched or mismatched low-voltage connection, length and type of low-voltage cable, waveform type, etc.

To avoid distorting measurements due to influence effects from nearby grounded or live objects, the reference divider needs to be placed at least 3 m away from surrounding objects and from the walls of the test room. An appropriately sized high-voltage connection could be used to link the impulse voltage injection point to the top terminal of the divider under test. This connection is considered an integral part of the divider during its characterization (scale factor measurement, step response, etc.).

The operations then proceed in six main steps:

- Waveform adjustment: These adjustments are carried out on the impulse voltage generator. Adjusting the generator may require placing some coupling capacitors in position. In such cases, it is important to keep these elements as far as possible from the high-voltage dividers. Particular attention is paid to eliminating superimposed oscillations on the front and any possible voltage overshoots. High-voltage connections should be kept as short as possible, as their inductive nature can lead to high-frequency oscillations, causing significant measurement errors if the bandwidth of the system under calibration is limited (oscillation filtering).
- Disturbance check: A disturbance test is performed following a procedure similar to that described in standard IEC 60060-2. The test is conducted on each measurement system. The disturbance level is defined as the ratio of the observed signal amplitude to the expected signal amplitude if the divider were connected normally to the high-voltage test circuit. This ratio must be less than 1%. If the disturbance level is unacceptable (≥1%), a new configuration must be considered for the transmission system (shielded or unshielded cables, groundings, cable positioning, etc.).
- Step response measurement: The measurements are complemented and validated in the time and frequency domain by analyzing the step response of the dividers. This step response analysis is intended to identify any anomalies in the time domain behavior of the dividers. The step generator is placed at the same height as the divider or in a rectangular configuration as described in Figure A2. Alternatively, a triangular configuration (with the generator placed on the ground) could be used for large dividers when the other configuration is not feasible. Data acquisition and processing are performed directly using the reference digitizer.

- Determination of the scale factor: The scale factor could be determined at low voltage by performing a frequency sweep from 60 Hz to 100 kHz at a voltage level higher than 100 V. The input and output voltages are measured using two calibrated reference voltmeters. This measurement is carried out under reference conditions, meaning the same configuration used during the disturbance testing must be strictly maintained. Care must be taken to ensure that the measurement instrumentation does not disturb these conditions by positioning it as far away as possible from the two dividers, preferably near the generator side (Figure A1). Both dividers should remain connected in parallel during this step.
- Digitizer calibration: The digital recorder is calibrated for the different types of waveforms to be measured. The reference waveforms are generated using a calibrated impulse generator.
- High-voltage measurement: Impulses are applied to both dividers connected in parallel, according to the setup shown in Figure A1. A direct comparison between the reference system and the system under calibration is performed.

## References

[1] (2010). High-Voltage Test Techniques—Part 1: General Definitions And Test Requirements.

[2] (2010). High-Voltage Test Techniques—Part 2: Measuring Systems.

[3] (2018). Instrument Transformers—Part 15: Additional Requirements for Voltage Transformers for DC Applications.

[4] (2017). High-Voltage Switchgear and Controlgear—Part 1: Common Specifications for Alternating Current Switchgear and Controlgear.

[5] (2017). General Requirements for the Competence of Testing and Calibration Laboratories.

[6] Saadeddine H., Agazar M., Fortune D. New reference systems for the calibration of HV impulses at LNE. Proceedings of the 19th International Congress of Metrology.

[7] Regulation (EU) 2024/573. https://eur-lex.europa.eu/eli/reg/2024/573/oj/eng.

[8] Hällström J., Mohamed Agazar S.E.C., Dedeoğlu S., Elg A.-P., Garcia T., Havunen J., Meisner J., Merev A., Özer S., Passon S. Design of a modular wideband high voltage reference divider. Proceedings of the Conference on Precision Electromagnetic Measurement (CPEM).

[9] Elg A.-P., Nieminen T., Klüss J., Passon S., Gerdinand F., Meisner J. A modular universal divider for calibration of UHVDC, and composite waves up to 1400 kV. Proceedings of the 23rd International Symposium on High Voltage Engineering (ISH 2023).

[10] Elg A.-P., Bergman A., Hällström J., Kharezy M., Nieminen T. (2015). Traceability and Characterization of a 1000 kV HVDC Reference Divider. IEEE Trans. Instrum. Meas..

[11] Rönnberg S., Bollen M. (2016). Power quality issues in the electric power system of the future. Electr. J..

[12] (2022). Power Cables with Extruded Insulation and their Accessories for Rated Voltages Above 150 kV (Um = 170 kV) up to 500 kV (Um = 550 kV)—Test Methods and Requirements.

[13] Franchi S.B. (2021). Recommendations for Testing DC Extruded Cable Systems for Power Transmission at a Rated Voltage up to and Including 800 kV.

[14] Metrology for Future Energy Transmission, 19ENG02 FutureEnergy. https://www.ptb.de/empir2020/futureenergy/home.

[15] de Lima V.R., Agazar M., Blanc I., Janin P.J., Vitório P., de Souza L.A.A., Ganvini O.W. Implementation of a high-voltage primary standard method using a capacitance bridge. Proceedings of the 29th Conference on Precision Electromagnetic Measurements (CPEM 2014).

[16] Blanc I. Measurements of high voltage impulses (400 kV): Implementation of a calibration test procedure. Proceedings of the Conference on Precision Electromagnetic Measurements Digest.

[17] Agazar M., Saadeddine H., Meisner J., Gerdinand F., Passon S., Pillet J.M. Determination of voltage dependence of capacitance of 100 kV and 300 kV compressed gas capacitors using the kinetic method. Proceedings of the 22nd International Symposium on High Voltage Engineering (ISH 2021), Hybrid Conference.

[18] Zaengl W. (1964). Das messen hoher, rasch verändlicher Stossspannungen.

[19] Feser K. (1974). Transient Behaviour of Damped Capacitive Voltage Dividers of Some Million Volts. IEEE Trans. Power Appar. Syst..

[20] (2008). Uncertainty of Measurement—Part 3: Guide to the Expression of Uncertainty in Measurement (GUM: 1995).

[21] Jones R.G. (2007). Report on Comparison EUROMET.EM-S14 (EUROMET Project 495): Measurements up to 100 kV DC. Metrologia.

[22] Simón P., Hällström J., Bergman A. (2022). Supplementary comparison for the traceability of AC high voltage reference measuring systems up to 200 kV (EURAMET.EM-S33). Metrologia.

[23] Hällström J., Elg A.-P., Havunen J., Garnacho F. (2020). Supplementary comparison EURAMET.EM-S42, comparison of lightning impulse (LI) reference measuring systems. Metrologia.

[24] Calibration and Measurement Capabilities—CMCs. https://www.bipm.org/kcdb.

[25] (2001). Instruments and Software Used for Measurement in High-Voltage Impulse Tests—Part 1: Requirements for instruments.

[26] (2011). Instruments and Software Used for Measurement in High-Voltage Impulse Tests—Part 1: Requirements for software for tests with impulse voltages and currents.

